# Editorial: Transforming crop resilience with multi-omics approaches

**DOI:** 10.3389/fpls.2026.1776693

**Published:** 2026-02-10

**Authors:** Ana Nikolić, Jelena Samardžić, Sofija Božinović

**Affiliations:** 1 Laboratory for Molecular Genetics and Physiology, Research Department, Maize Research Institute Zemun Polje, Belgrade, Serbia; 2 Laboratory for Plant Molecular Biology, Institute of Molecular Genetics and Genetic Engineering, University of Belgrade, Belgrade, Serbia; 3 Breeding Department, Maize Research Institute Zemun Polje, Belgrade, Serbia

**Keywords:** abiotic stress, biotic stress, crop resilience, multi-omics, precision breeding, sustainable agriculture

The Research Topic Transforming Crop Resilience with Multi-Omics Approaches highlights how the integration of diverse -omics technologies is reshaping strategies for sustainable crop production. Rather than relying primarily on physical or chemical interventions, the studies collected in this Topic emphasize a paradigm shift toward genetically encoded resilience, enabling crops to withstand environmental stresses under increasingly variable climatic conditions. Together, these contributions illustrate how multi-omics approaches accelerate biological insight and translate molecular knowledge into practical breeding solutions for “climate-smart” agriculture.

One study demonstrates how multi-omics can unravel complex interactions at the plant–soil interface. Continuous tobacco cropping imposes abiotic stress, leading to degradation of soil health and the microbiome, nutrient imbalances, and yield loss. Liu et al. integrated metagenomics and metabolomics to analyze the effects of intercropping tobacco with maize and soybean on the rhizosphere to mitigate negative impacts of persistent tobacco cultivation. Researchers identified a “gene metabolite–function” mechanism that remodels the rhizosphere. The process enables enrichment of soil metabolites and activates genetic pathways that allow tobacco plants to “breathe” and absorb nutrients more effectively at the cellular level, even in depleted soil. Hence, application of multiomics strategies enhances the likelihood of gaining better understanding of processes within the rhizosphere, providing reliable information in a brief timeframe and fostering the achievement of sustainable agriculture by improving crop tolerance to abiotic stress.

Several contributions focus on stress resilience in reproductive and vegetative tissues, emphasizing that stress tolerance is often organ-specific and dynamically regulated. Sun et al. investigated cold resistance mechanisms in pear reproductive organs under spring frost conditions. Transcriptomic analyses provided a molecular blueprint of how pear plants actively respond to cold stress through reprogramming of gene expression and metabolism, indicating an active survival strategy. Key pathways involved included flavonoid biosynthesis, the phenylpropanoid pathway, and starch and sucrose metabolism. These findings suggest that marker-assisted selection or gene editing could be used to develop frost-tolerant pear varieties, reducing reliance on energy-intensive orchard heating while also shortening breeding timelines. Together, these results point toward genomic-based solutions that lower production costs and environmental impact.

Zhang et al. aimed to identify the genetic basis of drought tolerance in *Brassica juncea* L. (brown mustard) to facilitate the breeding of more resilient varieties using a multifaceted omics approach. By integrating GWAS, co-expression network analysis, and transcriptomics, the study elucidates the genetic architecture underlying drought tolerance. This multi-layered framework reveals coordinated stress responsive gene networks and key genomic regions associated with adaptive responses, providing a robust basis for accelerating precision breeding strategies. Ultimately, the implementation of integrative -omics approaches can shorten breeding timelines, improve target selection, and enhance understanding of plant survival strategies, thereby supporting the development of climate-smart genotypes with tangible economic benefits.

Similar integrative approaches are applied in upland cotton by Sun et al. The goal of the study was to map the regulatory network of drought response using transcriptomics and metabolomics and to identify where they overlapped. It is important to prove that both the specific gene is active (transcriptomics) and that the specific gene product (metabolomics) is present for these multi-omic findings to be useful for breeding. Additionally, transcriptomics allows for the discovery of candidate genes that confer drought tolerance, serving as a resource for MAS and gene editing and thus enabling precision breeding and economic gains.

The research by Tianxiao et al. provides comprehensive multi-omics atlas (integrated epigenomics and 3D genomics approach - ChIP-seq, Hi-C, histone modification, ATAC-seq) of how the GmLUX transcription factor regulates flowering in soybeans. By advancing past basic gene identification this research uncovers a complex dimension of “epigenetic regulation” affecting crop growth. The study exemplifies the transition from broad traditional breeding toward precision breeding informed by regulatory and epigenetic mechanisms.

Liu et al. investigated resistance mechanisms in Sea Island cotton (*Gossypium barbadense*) against Fusarium wilt by integrating bulk tissue transcriptomics with single-nucleus RNA sequencing. While bulk RNA-seq captures a generalized stress response at the tissue level, single-nucleus analysis resolves defense responses at cellular resolution, revealing a localized gene module that functions as the first line of defense in the root outer layers. The integration of cell-resolved transcriptomics with network and spatial analyses narrows broad stress signatures to a small set of early-response genes with cell-specific activation. This cell-type-specific insight enables highly targeted resistance strategies and highlights the potential for precision breeding approaches that activate defense pathways only at the site and timing of pathogen infection.

Collectively, the articles in this Research Topic underscore how multi-omics integration—spanning soil microbiomes, whole tissues, and single cells—enables a unified and mechanistic understanding of crop resilience. These approaches shorten breeding timelines, improve selection accuracy, and reduce dependence on environmentally costly management practices. Looking forward, the continued integration of multi-omics with high-throughput phenotyping, computational modeling, and artificial intelligence will be essential for translating molecular complexity into robust, scalable solutions for sustainable agriculture.

